# Metabolomics for Agricultural Waste Valorization: Shifting Toward a Sustainable Bioeconomy

**DOI:** 10.3389/fpls.2022.938480

**Published:** 2022-06-27

**Authors:** Gholamreza Khaksar, Mongkon Sirijan, Nithiwat Suntichaikamolkul, Supaart Sirikantaramas

**Affiliations:** ^1^Center of Excellence for Molecular Crop, Department of Biochemistry, Faculty of Science, Chulalongkorn University, Bangkok, Thailand; ^2^Faculty of Agriculture Natural Resources and Environment, Naresuan University, Phitsanulok, Thailand; ^3^Omics Sciences and Bioinformatics Center, Chulalongkorn University, Bangkok, Thailand

**Keywords:** bioactive compound, metabolomics, organic agricultural waste, valorization, value-added product

## Abstract

Agriculture has been considered as a fundamental industry for human survival since ancient times. Local and traditional agriculture are based on circular sustainability models, which produce practically no waste. However, owing to population growth and current market demands, modern agriculture is based on linear and large-scale production systems, generating tons of organic agricultural waste (OAW), such as rejected or inedible plant tissues (shells, peels, stalks, etc.). Generally, this waste accumulates in landfills and creates negative environmental impacts. The plant kingdom is rich in metabolic diversity, harboring over 200,000 structurally distinct metabolites that are naturally present in plants. Hence, OAW is considered to be a rich source of bioactive compounds, including phenolic compounds and secondary metabolites that exert a wide range of health benefits. Accordingly, OAW can be used as extraction material for the discovery and recovery of novel functional compounds that can be reinserted into the production system. This approach would alleviate the undesired environmental impacts of OAW accumulation in landfills, while providing added value to food, pharmaceutical, cosmetic, and nutraceutical products and introducing a circular economic model in the modern agricultural industry. In this regard, metabolomics-based approaches have gained increasing interest in the agri-food sector for a variety of applications, including the rediscovery of bioactive compounds, owing to advances in analytical instrumentation and data analytics platforms. This mini review summarizes the major aspects regarding the identification of novel bioactive compounds from agricultural waste, focusing on metabolomics as the main tool.

## Introduction

For centuries, the agricultural industry has been vital for providing food and materials to humankind. Traditional and local agriculture utilize available plants and resources (water, soil, land, etc.) in a sustainable way, ensuring the subsistence of the local community. Practically no waste is produced in these approaches as waste and unexploited products are utilized further as fertilizers, which are easily absorbed by soils (Harris and Hillman, 2014). In the wine production industry, the generated grape marc would be further utilized in the production of other alcohols, and the final product would be used to fertilize the soil (Nerantzis and Tataridis, 2006). However, population growth and its consequent need to produce large amounts of food, as well as globalization and the pursuit of individual economic benefits have promoted the emergence of a linear-producing modern agricultural system. Unlike traditional and local agriculture, which are based on circular sustainability models, the modern system aims to meet global demand by increasing the profitability of production. To achieve this aim, modern agriculture overexploits natural resources by using the soil extensively along with large amounts of water and energy, applying pesticides to eliminate insects, and choosing monocultures over mixed production (Rockström et al., 2017; Ramankutty et al., 2018; Duque-Acevedo et al., 2020). The world population is predicted to reach ~10 billion by 2050 (data from Department of Economic and Social Affairs, United Nations (UN), n.d., Food and Agriculture Organization (FAO), n.d.). Accordingly, agricultural production also needs to grow, albeit in a sustainable way (Ramankutty et al., 2018). However, a major drawback of this modern system is the increased production of organic agricultural waste (OAW) from crops (Gustavsson et al., 2011), including rejected or inedible plant tissues such as pruning, fruit trimming, shelling or forestall residues, and food processing wastes such as rice husk and wheat straw. Notably, fruit pulp is also considered a major OAW because fruit juice production generates tons of squeezed pulp. In addition, huge quantities of fruit pulp may be rejected owing to post-harvest loss, as is commonly seen for climacteric fruits that possess a strikingly limited shelf life after harvesting. Generally, OAWs that are not further utilized accumulate uncontrollably in landfills. If poorly managed, the accumulated OAW would generate various biotic and abiotic by-products that would negatively impact the environment, health, and economy (El-Haggar, 2007; Nagendran, 2011; He et al., 2019). Moreover, such landfills generate considerable amounts of methane, nitrous oxide, sulfur dioxide, and smoke when the OAW is burned in open air, a practice which is common in many landfills and leads to atmospheric pollution with significant emissions of carbon dioxide (Wang et al., 2019).

## Toward a Sustainable Bioeconomy Using Organic Agricultural Waste

The question arises as to why OAW is not reutilized. Different reutilization approaches for these residues have been described, including as animal feed and subjection to anaerobic digestion and composting. Although the effectiveness of these strategies has been described by several studies, such as those demonstrating the beneficial effects of using OAW as fertilizer (Sud et al., 2008; Meng et al., 2017), OAWs continue to accumulate. The reason for this is profitability. For instance, farmers are not willing to risk replacing synthetic fertilizers, which deliver a precise quantity of nutrients, with OAW, which provides an imprecise quantity (Innes, 2013). Moreover, these strategies do not typically generate significant economic value (Garcia-Garcia et al., 2019). Therefore, a shift toward a more sustainable approach is vital. Over the last decades, the development of novel value-added products based on the exploitation of bioactive compounds from OAW has gained considerable interest, which makes OAW a suitable feedstock for valorization. The plant kingdom is extensively rich in metabolic diversity, harboring over 200,000 structurally distinct metabolites (Wurtzel and Kutchan, 2016) that are naturally present in plants, especially under stress and/or damage conditions, which also magnifies their presence in OAWs. Hence, OAW is a rich source of bioactive compounds, including phenolic compounds (PCs) and secondary metabolites, which exert a wide range of health benefits such as antioxidant, anti-cancer, anti-inflammatory, cardioprotective, anti-microbial, and anti-allergenic activities (Coman et al., 2020; Jimenez-Lopez et al., 2020). PCs are a large group of secondary metabolites generated by plants in response to multiple environmental stimuli. Owing to the numerous health-beneficial properties associated with PCs and their abundance in OAW, PCs from OAW are increasingly attracting industrial interest. Moreover, since some of these compounds are difficult and/or expensive to synthesize, their availability from OAW makes chemical synthesis unnecessary (Burri et al., 2017; Jimenez-Lopez et al., 2020).

The importance of converting OAW into value-added products has been incorporated into various market sectors. According to a recent study, the market value of agricultural waste products peaked at USD 63.3 billion in the beverage industry, followed by USD 48 billion in the medical industry and approximately USD 46 billion in the food and consumer goods sector (Beltrán-Ramírez et al., 2019). Taken together, the valorization of OAW generates a significant economic value by increasing income per harvest and improving the livelihood of the local communities, while reducing the excessive costs for waste disposal and minimizing the carbon footprint (Lucarini et al., 2018; Singh et al., 2019).

## Shift Toward Sustainable Agriculture: Valorization of OAW as a Potential Source of Bioactive Compounds

Moving toward a systemic, circular model of “reuse, recycle, and regenerate” is vital for developing a sustainable agricultural industry. In this context, OAW biomass should be considered a sustainable resource rather than a waste product. OAW valorization is based on the concept that any residual material or by-product can be used as an extraction material, and the recovered bioactive compounds be reinserted into the production chain. The reutilization of these functional compounds not only represents various potential applications, including in the preparation of functional foods, food and feed additives, and nutraceutical and cosmeceutical products, but also alleviates certain negative effects of OAW accumulation in landfills, thus representing a favorable measure for the environment.

The extraction of these functional bioactive compounds is an important aspect of OAW valorization both in the context of economic benefit, owing to the recovery of valuable compounds, and in the context of waste detoxification, owing to the removal of some compounds which could be undesirable in subsequent biological post-treatments (Serrano et al., 2017; Negro et al., 2018). Over the last few decades, researchers have focused on optimizing the extraction processes. Different parameters have been investigated to optimize the extraction yields of bioactive compounds available in OAW (Kareem and Rahman, 2013; Dorta et al., 2014; Wong et al., 2014). Table 1 summarizes the different OAWs from various crops, the amounts generated in Asia (tons/year) as of 2020, the bioactive compounds extracted from them, and the main analytical platforms utilized according to the literature published in the last 5 years (2017 until now).

**Table 1:** List of organic agricultural wastes (OAWs) from various crops, the major bioactive compounds identified and/or extracted from them, main analytical platform utilized, and recent research studies (2017–present) in this area.

Crop	Agricultural waste	Amount of waste produced in Asia (tons/year) in 2020^*^	Bioactive compounds	Analytical platform	References
Almond	Skin from seed	648,111	Catechin, kaempferol, isorhamnetin, naringenin, quercetin	HPLC	Chen et al., 2019
Apple	Pomace, seed, peel	55,707,264	Anthocyanins, catechin, caffeic acid, phloretin glycosides, quercetin glycosides, rutin	HPLC	Călinoiu et al., 2017; Gunes et al., 2019; Nile et al., 2019
Avocado	Peel, seed	943,327	Catechin, chlorogenic acid, cyanidin, epicatechin, gallic acid, hydroxybenzoic acid, procyanidins, 1-caffeoylquinic acid, 3-glucosidecitric acid, 3-O-p-coumaroylquinic acid, 4-caffeoylquinic acid	LC–MS/MS, HPLC	Tremocoldi et al., 2018
Banana	Peel, stalk, pulp	64,730,743	Anthocyanins, auroxanthin, catecholamine, cyaniding, delphinidin, gallocatechin, hydroxycinnamic, flavonoids, isolutein, lutein, neoxanthin, α-carotene, β-carotene, β-cryptoxanthin	LC–MS/MS	Kraithong and Issara, 2021
Barley	Husk	25,516,523	Catechins, flavonoids, gallocatechin, cis-ferulate, trans-ferulate	HPLC	Nigam, 2017
Carrot	Peel	26,126,853	Anthocyanidin, carotenoids, α-carotene, β-carotene	HPLC	Gulsunoglu et al., 2019
Cauliflower	Stem, leaves	No data	Caffeic acid, ferulate, glucoiberin	HPLC	Xu et al., 2017
Cocoa	Skin, husk, shell	777,259	Apigenin, catechin, epicatechin	LC–MS/MS, HPLC	Campos-Vega et al., 2018
Coffee	Cherry pulp	No data	Anthocyanins, caffeic acid, chlorogenic acid, di-caffeoylquinic acid	HPLC	Heeger et al., 2017
Corn	Bran	365,305,747	Anthocyanins, caffeic acid, ferulate, *p*-coumaric acid	LC–MS/MS, HPLC	Luna-Vital et al., 2017
Date	Pulp, seed	370,583,855	Phenolic acids, flavonols, fatty acids, sphingolipids, steroids	LC–MS, NMR	Otify et al., 2019
Durian	Peel, pulp, rind, seed	1,111,928 (in Thailand)	Glutathione, γ-glutamylcysteine, pyridoxamine, cysteine, leucine	CE-MS, HPLC, GC–MS, HPAEC-PAD	Pinsorn et al., 2018; Cui et al., 2021; Panpetch and Sirikantaramas, 2021; Ramli et al., 2021; Sangpong et al., 2021
Grape	Stalk, seed, pulp	29,824,812	Anthocyanins, caffeic acid, catechins, coumarate, epicatechin	HPLC–MS/MS, HPLC	Mattos et al., 2017
Grapefruit	Peel, pulp, seed	No data	Neohesperidosides, naringenin	HPIEC, LC–MS, GC–MS	Ahmed et al., 2019; Fernandez-Fernandez et al., 2020; Dorado et al., 2021
Lemon	Seed, peel, pulp	920,592	Apigenin-6, caffeic acid, coumarate, ferulate	LC–MS, HPLC	Sharma et al., 2017; Long and Mohan, 2021
Mango	Skin, pulp, seed	39,742,461	Flavonoids, gallates, hydrolysable tannins, methyl gallate, phenolics	LC–MS	Baddi et al., 2018; Bernal-Mercado et al., 2018; Wall-Medrano et al., 2020
Orange	Peel, seed, pulp	28,366,264	Caffeic acid, chrologenic acid, cinnamic, ferulate, *p*-Coumaric acid, heperetin, hesperidin, hesperetin-7-O-rutinoside, naringenin-7-O-rutinoside	GC	Pacheco et al., 2018
Papaya	Seed, peel	7,814,260	Carotene, cryptoxanthin, lutein	HPLC	Siddique et al., 2018
Pineapple	Stem, pulp, peel	12,500,507	Catechin, epicatechin, ferulate, gallic acid, phenolics	LC–MS, HPLC	Campos et al., 2020
Pomegranate	Pulp, seed, peel	No data	Anthocyanins, flavonoids, gallic acid, punicalagin	HPLC	Sandhya et al., 2018; Meselhy et al., 2020
Potato	Peel, tuber, leaf	178,599,864	Anthocyanin, caffeic acid, carotenoid, lutein, 5-O-caffeoylquinic acid, 3-O-caffeoylquinic acid, 4-O-caffeoylquinic acid, 4,5-di-O-caffeoylquinic acid, 3,5-di-O-caffeoylquinic acid, 3,4-di-O-caffeoylquinic acid, 3,4,5-tri-O-caffeoylquinic acid	HPAEC-PAD	Scharf et al., 2020
Rice	Husk, straw, bran	676,610,485	Anthocyanins, caffeic acid, ferulate, niacin, pantothenic, pyridoxine, phytosterols, tricin, tocopherols, tocotrienols, thiamine	HPLC, LC–MS	Perez-Ternero et al., 2017; Bodie et al., 2019; Peanparkdee and Iwamoto, 2019
Soybean	Husk	33,560,440	chlorogenic acid, ferulate, gallic acid	HPLC	Carneiro et al., 2020
Sweet potato	Peel, tuber, leaf	55,979,599	Anthocyanin, caffeic, lutein, 5-O-caffeoylquinic acid, 3-O-caffeoylquinic acid, 4-O-caffeoylquinic acid, 4,5-di-O-caffeoylquinic acid, 3,5-di-O-caffeoylquinic acid, 3,4-di-O-caffeoylquinic acid, 3,4,5-tri-O-caffeoylquinic acid	HPLC	Akoetey et al., 2017
Tomato	Peel, pulp, seed	116,993,632	Caffeic acid, chlorogenic acid, ferulate, β-carotene, lycopene	HPLC, LC–MS/MS	Szabo et al., 2018; Coelho et al., 2019; Lu et al., 2019
Wheat	Bran	347,921,349	Caffeic acid, ferulate, gallic acid, *p*-coumaric acid	HPLC, LC–MS/MS	Seifdavati et al., 2021; Sisti et al., 2021

* *retrieved from https://www.fao.org/faostat/en/#home*.

## Metabolomics in Agri-Food Sector: Current Practices for Valorization of OAW

Metabolomics is the comprehensive characterization of small molecules or metabolites present in a biological sample. Owing to the development of chemometrics and advanced analytical platforms, metabolomics has deepened our understanding of various metabolomic and pathway networks (Hollywood et al., 2006). Numerous high-throughput analytical platforms, including liquid chromatography and gas chromatography–mass spectrometry (LC–MS and GC–MS), and nuclear magnetic resonance (NMR) spectroscopy, have been extensively utilized for this purpose (Johanningsmeier et al., 2016). Metabolomics studies use untargeted or targeted approaches, and the selection of the analytical approach depends mainly on the research question and expected outcomes. Targeted analyses focus on a class of metabolites of interest based on our pre-existing knowledge. However, untargeted analyses utilize unbiased metabolite fingerprinting to profile the global metabolome of diverse chemical classes of metabolites associated with various known and/or unknown pathways (Scalbert et al., 2009; Patti et al., 2012). As shown in Table 1, high-performance liquid chromatography (HPLC) has been extensively utilized as the main analytical platform for the identification and/or discovery of various bioactive compounds from OAW using a targeted approach. However, over the past few years, a combination of both targeted and untargeted approaches (HPLC coupled with LC–MS and/or GC–MS) has been utilized to obtain a complete profile of the metabolites present in OAWs (as seen in Table 1).

Over the past few decades, metabolomics has been extensively applied to the valorization of different OAWs from various crops. Owing to recent advances in analytical instrumentation and data analytics platforms (Putri et al., 2013; Rubert et al., 2015), metabolomics-based approaches have gained significant interest in the agri-food sector for the identification and/or rediscovery of diverse high-value bioactive compounds, especially PCs, from OAWs. Over 10,000 different PC structures with diverse natures are currently known, the most well-known of which include phenolic acids, flavonoids, and tannins (Kennedy and Wightman, 2011). These exist naturally in various concentrations in different plant parts, from roots to shoots, as well as in fruits. Accordingly, they are also present in OAWs. Recently, numerous studies have focused on the research and development of natural compounds as substitutes for synthetic additives because synthetic substances are strongly associated with various health risks, such as the appearance of allergies or even carcinogenesis (Zheng and Wang, 2001). In this context, OAWs are considered as suitable source materials for the extraction of numerous natural bioactive compounds, such as PCs, and metabolomics can be considered as an ideal approach for the identification and/or rediscovery of these compounds from OAWs.

Numerous value-added products have been generated in the food, pharmaceutical, cosmetic, and nutraceutical industries by reinserting these high-value compounds into the production chain, as in a circular economic model. Typical examples include thickening, gelling, and food stabilizing agents from tomato and citrus waste (John et al., 2017; Morales-Contreras et al., 2017); food preservatives (meat and oil product preservatives), food stabilizers, and bactericidal agents from potato peel (Sampaio et al., 2020); essential oils with anti-cancer agents from orange waste (Yang et al., 2017); biobutanol from rice husk (Quispe et al., 2017); hydrogel from durian rind (Cui et al., 2021); and single-cell protein (SCP) from corn stover and orange peel (Diwan et al., 2018).

These value-added products, produced through OAW valorization, generate significant economic value. However, further research and development is vital to fully convert the still-evolving valorization process into a sustainable approach. By integrating metabolomics into this process, we can gain a deeper understanding of the metabolic profiles of OAWs, and this can further promote the valorization process and add greater value to such products. To the best of our knowledge, only a few studies have utilized metabolomics to fully profile the metabolome of OAW and enhance the value of such products. These include studies on the production of pineapple wine and vinegar from pineapple peel and pulp (Roda et al., 2017), essential oils from the aerial parts of plants belonging to the genus *Lavandula*, mainly *L. angustifolia* (LA) and *L.* × *intermedia* (LI; Truzzi et al., 2022), supplements with therapeutic applications from *Passiflora mollissima* seeds (Ballesteros-Vivas et al., 2020), and functional foods and nutraceuticals from bean (*Vicia faba* L.) by-products (Abu-Reidah et al., 2017).

**Figure 1:**
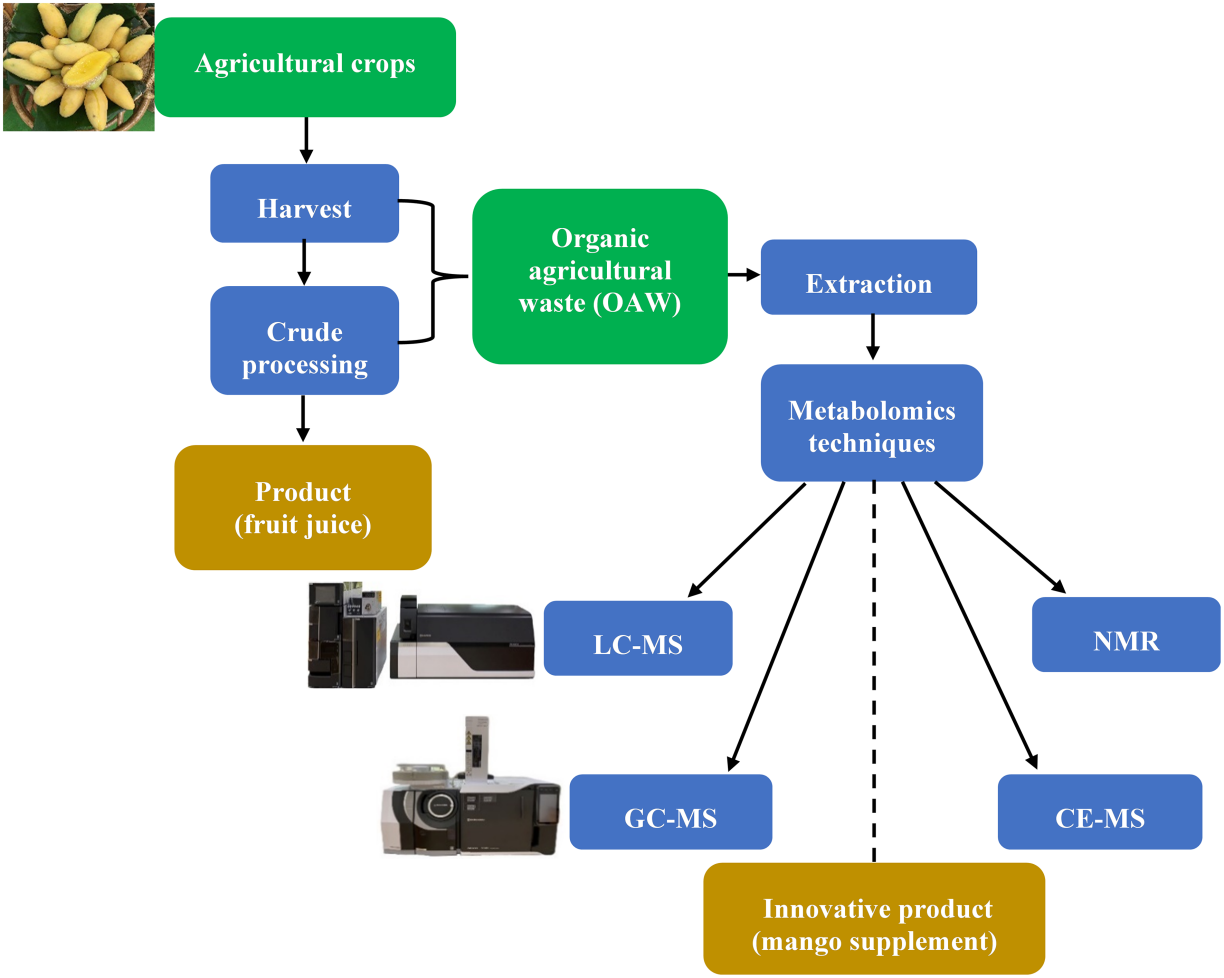
A schematic overview of the application of metabolomics in OAW valorization in a circular economy concept. The dotted line represents the multiple steps that might be needed to generate the final product.

Taken together, the recovery of functional bioactive compounds can be achieved by obtaining products that can be reinserted into the economy as new raw materials within a circular and sustainable bioeconomy. Figure 1 presents a schematic overview of the application of metabolomics in OAW valorization using a circular economy concept. OAW was considered as the input material for the valorization process in this overview. To gain a deeper understanding of the metabolic profile of OAW, we can take advantage of metabolomics to identify and/or rediscover bioactive compounds that can then be reinserted into the production chain to generate value-added products. Notably, for non-edible wastes such as peels and seeds, more studies, including toxicity tests and/or animal model studies, are needed to ensure their safety.

## Conclusion and Future Perspective

Owing to population growth and current market demands, modern agricultural systems are linear in nature and generate millions of tons of OAW. These wastes accumulate in landfills and create adverse environmental impacts. Since OAWs are rich in bioactive compounds, including secondary metabolites and PCs, which have various health benefits, their valorization will provide us with numerous exploitable economic, environmental, and social opportunities. To develop a circular and sustainable bioeconomy, OAW can be used as an extraction material, and the recovered bioactive compounds can be reinserted into the production chain. In this regard, metabolomics-based approaches have gained increasing interest in the agri-food sector for the identification and/or rediscovery of these bioactive compounds. Additionally, OAW valorization can be used as a powerful and effective approach for tackling current global issues, including food shortages, waste disposal, and landfill reserves. However, further investigation is still vital to optimize extraction techniques to obtain increased product yields in an eco-friendly and economical manner. Moreover, further developments are required to fully integrate the currently evolving valorization system into a sustainable and efficient industrial tool. In this context, metabolomics can be utilized as a powerful tool to obtain a complete metabolic profile of OAWs. An important risk factor in this process could be the presence of contamination from chemicals in the crop residues owing to excessive use of pesticides and synthetic fertilizers, which should be taken into consideration. There is a dilemma among gardeners and/or orchard owners whether to use pesticides and chemical fertilizers as much as needed to avoid any yield loss and gain profit from higher quality products, or to minimize or, if possible, avoid the use of pesticides and provide the valorization industry with non-contaminated OAWs and gain profit from the OAW valorization process. For the latter to occur, we need to increase public awareness regarding the importance and need to shift toward a circular and sustainable bioeconomy in which OAW is considered a natural resource for the valorization process. In addition, more companies should dedicate themselves to the valorization of OAW and production of value-added products. Taken together, it can be concluded that, although metabolomics can be used as an effective tool to improve the valorization potential of OAWs, the question as to which approach to follow remains open-ended.

## Author Contributions

SS: conceptualization and supervision. GK and SS: writing—original draft preparation. GK, MS, NS, and SS: writing—review and editing. All authors contributed to the article and approved the submitted version.

## Funding

Research in our laboratory was supported by Chulalongkorn University Fund (GRU 6407023008-1).

## Conflict of Interest

The authors declare that the research was conducted in the absence of any commercial or financial relationships that could be construed as a potential conflict of interest.

## Publisher’s Note

All claims expressed in this article are solely those of the authors and do not necessarily represent those of their affiliated organizations, or those of the publisher, the editors and the reviewers. Any product that may be evaluated in this article, or claim that may be made by its manufacturer, is not guaranteed or endorsed by the publisher.

## References

[ref01] Abu-Reidah I. M. Arráez-Román D. Warad I. Fernández-Gutiérrez A. Segura-Carretero A. (2017). UHPLC/MS_2_-based approach for the comprehensive metabolite profiling of bean (*Vicia faba L.*) by-products: A promising source of bioactive constituents. Food Res. Int. 93, 87–96. doi: 10.1016/j.gecco.2020.e00902 28290284

[ref1] Ahmed S. Rattanpal H. S. Gul K. Dar R. A. Sharma A. (2019). Chemical composition, antioxidant activity and GC-MS analysis of juice and peel oil of grapefruit varieties cultivated in India. J. Integr. Agric. 18, 1634–1642. doi: 10.1016/S2095-3119(19)62602-X

[ref2] Akoetey W. Britain M. M. Morawicki R. O. (2017). Potential use of byproducts from cultivation and processing of sweet potatoes. Cienc. Rural 47:e20160610. doi: 10.1590/0103-8478cr20160610

[ref3] Baddi J. Vijayalakshmi D. Kapale M. (2018). Extraction of total polyphenols and dietary fiber from mango peel - as potential sources of natural phytonutrients. Int. J. Curr. Microbiol. App. Sci. 7, 1196–1205. doi: 10.20546/ijcmas.2018.705.146

[ref02] Ballesteros-Vivas D. Alvarez-Rivera G. León C. Morantes S. J. Ibánez E. Parada-Alfonso F.. (2020). Foodomics evaluation of the anti-proliferative potential of Passiflora mollissima seeds. Food Res. Int. 130:108938. doi: 10.1016/j.foodres.2019.108938 32156385

[ref4] Beltrán-Ramírez F. Orona-Tamayo D. Cornejo-Corona I. González-Cervantes J. L. N. de Jesús Esparza-Claudio J. Quintana-Rodríguez E. (2019). “Agro-industrial waste revalorization: the growing biorefinery,” in Biomass for Bioenergy-Recent Trends and Future Challenges. ed. Abomohra A. E.-F. (London, UK: IntechOpen), 1–20.

[ref5] Bernal-Mercado A. T. Acevedo-Hernandez C. Silva-Espinoza B. A. Cruz-Valenzuela M. R. Gonzalez-Aguilar G. A. Nazzaro F.. (2018). Antioxidant and antimicrobial capacity of phenolic compounds of mango (*Mangifera indica* L.) seed depending upon the extraction process. J. Med. Plants Prod. 7, 209–219. doi: 10.22092/jmpb.2018.118149

[ref6] Bodie A. R. Micciche A. C. Atungulu G. G. Rothrock M. J. Jr. Ricke S. C. (2019). Current trends of rice milling byproducts for agricultural applications and alternative food production systems. Front. Sustain. Food Syst. 3:47. doi: 10.3389/fsufs.2019.00047

[ref7] Burri S. C. M. Ekholm A. Håkansson Å. Tornberg E. Rumpunen K. (2017). Antioxidant capacity and major phenol compounds of horticultural plant materials not usually used. J. Funct. Foods 38, 119–127. doi: 10.1016/j.jff.2017.09.003, PMID: 29129982 PMC5666123

[ref8] Călinoiu L. F. Mitrea L. Precup G. Bindea M. Rusu B. Dulf F. V.. (2017). Characterization of grape and apple peel wastes’ bioactive compounds and their increased bioavailability after exposure to thermal process. Bull. Univ. Agric. Sci. Veter-Med. Cluj-Napoca. Food Sci. Technol. 74:80. doi: 10.15835/buasvmcn-fst:0028

[ref9] Campos D. A. Ribeiro T. B. Teixeira J. A. Pastrana L. Pintado M. M. (2020). Integral valorization of pineapple (*Ananas comosus* L.) by-products through a green chemistry approach towards added value ingredients. Foods. 9:60. doi: 10.3390/foods9010060, PMID: 31936041 PMC7022615

[ref10] Campos-Vega R. Nieto-Figueroa K. H. Oomah B. D. (2018). Cocoa (*Theobroma Cacao* L.) pod husk: renewable source bioactive compounds. Trends Food Sci. Technol. 81, 172–184. doi: 10.1016/j.tifs.2018.09.022

[ref11] Carneiro A. M. Moreira E. A. Bragagnolo F. S. Borges M. S. Pilon A. C. Rinaldo D.. (2020). Soya agricultural waste as a rich source of isoflavones. Food Res. Int. 130:108949. doi: 10.1016/j.foodres.2019.108949, PMID: 32156391

[ref12] Chen C. Y. O. Milbury P. E. Blumberg J. B. (2019). Polyphenols in almond skins after blanching modulate plasma biomarkers of oxidative stress in healthy humans. Antioxidants. 8:95. doi: 10.3390/antiox8040095, PMID: 30974789 PMC6523744

[ref13] Coelho M. Pereira R. Rodrigues A. S. Teixeira J. A. Pintado M. E. (2019). Extraction of tomato by-products’ bioactive compounds using ohmic technology. Food Bioprod. Process. 117, 329–339. doi: 10.1016/j.fbp.2019.08.005

[ref14] Coman V. Teleky B. E. Mitrea L. Martau G. A. Szabo K. Calinoiu L. F.. (2020). “Bioactive potential of fruit and vegetable wastes,” in Advances in Food and Nutrition Research. ed. Toldra F. (Cambridge: Academic Press), 157–225. 10.1016/bs.afnr.2019.07.001 32035596

[ref15] Cui X. Lee J. Ng K. R. Chen W. N. (2021). Food waste durian rind-derived cellulose organohydrogels: toward anti-freezing and antimicrobial wound dressing. ACS Sustain. Chem. Eng. 9, 1304–1312. doi: 10.1021/acssuschemeng.0c07705

[ref16] Department of Economic and Social Affairs, United Nations (UN) (n.d.) Available at: https://www.un.org/en/desa/world-population-projected-reach-98-billion-2050-and-112-billion-2100 (Accessed 5 May 2022).

[ref03] Diwan B. Parkhey P. Gupta P. (2018). From agro-industrial wastes to single cell oils: a step towards prospective biorefinery. Folia Microbiol. 63, 547–568. doi: 10.1007/s12223-018-0602-7 29687420

[ref17] Dorado C. Cameron R. G. Manthey J. A. Bai J. Ferguson K. L. (2021). Analysis and potential value of compounds extracted from star ruby, rio red, and ruby red grapefruit, and grapefruit juice processing residues *via* steam explosion. Front. Nutr. 8:691663. doi: 10.3389/fnut.2021.691663, PMID: 34589509 PMC8473638

[ref18] Dorta E. González M. Lobo M. G. Sánchez-Moreno C. de Ancos B. (2014). Screening of phenolic compounds in by-products extracts from mangoes (*Mangifera indica* L.) by HPLC-ESI-QTOF-MS and multivariate analysis for use as food ingredients. Food Res. Int. 57, 51–60. doi: 10.1016/j.foodres.2014.01.012

[ref04] Duque-Acevedo M. Belmonte-Ureña L. J. Cortés-García F. J. Camacho-Ferre F. (2020). Agricultural waste: Review of the evolution, approaches and perspectives on alternative uses. Glob. Ecol. Conserv., 22:e00902.

[ref19] El-Haggar S. M. (2007). “Sustainable Development and Environmental Reform,” in Sustainable Industrial Design and Waste Management. eds. T.-S. I. D. S. M. B. El-Haggar W. M. (Oxford: Elsevier), 125–148.

[ref20] Fernandez-Fernandez A. M. Dellacassa E. Medrano-Fernandez A. Del Castillo M. D. (2020). “Citrus waste recovery for sustainable nutrition and health,” in Food Wastes and By-Products: Nutraceutical and Health Potential. eds. Campos-Vega R. Oomah B. D. Vergara-Castaneda H. A. (Hoboken, New Jersey, U.S.: Blackwell Publishing), 193–222.

[ref21] Food and Agriculture Organization (FAO) (n.d.). Available at: https://www.fao.org/faostat/en/#home (Accessed 7 February 2022).

[ref22] Garcia-Garcia G. Stone J. Rahimifard S. (2019). Opportunities for waste valorisation in the food industry – a case study with four UK food manufacturers. J. Clean. Prod. 211, 1339–1356. doi: 10.1016/j.jclepro.2018.11.269

[ref23] Gulsunoglu Z. Karbancioglu-Guler F. Raes K. Kilic-Akyilmaz M. (2019). Soluble and insoluble-bound phenolics and antioxidant activity of various industrial plant wastes. Int. J. Food Prop. 22, 1501–1510. doi: 10.1080/10942912.2019.1656233

[ref24] Gunes R. Palabiyik I. Toker O. S. Konar N. Kurultay S. (2019). Incorporation of defatted apple seeds in chewing gum system and phloridzin dissolution kinetics. J. Food Eng. 255, 9–14. doi: 10.1016/j.jfoodeng.2019.03.010

[ref25] Gustavsson J. Cederberg C. Sonesson U. Van Otterdijk R. Meybeck A. (2011). “Global food losses and food waste.” in *Proceedings of the Save Food Congress FAO, 2011*. Rome, Italy.

[ref26] Harris D. R. Hillman G. C. (2014). Foraging and Farming: The Evolution of Plant Exploitation. London, UK: Routledge.

[ref27] He K. Zhang J. Zeng Y. (2019). Knowledge domain and emerging trends of agricultural waste management in the field of social science: a scientometric review. Sci. Total Environ. 670, 236–244. doi: 10.1016/j.scitotenv.2019.03.184, PMID: 30903897

[ref28] Heeger A. Kosińska-Cagnazzo A. Cantergiani E. Andlauer W. (2017). Bioactives of coffee cherry pulp and its utilisation for production of cascara beverage. Food Chem. 221, 969–975. doi: 10.1016/j.foodchem.2016.11.067, PMID: 27979301

[ref29] Hollywood K. Brison D. R. Goodacre R. (2006). Metabolomics: current technologies and future trends. Proteomics 6, 4716–4723. doi: 10.1002/pmic.200600106 16888765

[ref30] Innes R. (2013). “Economics of agricultural residuals and overfertilization: chemical fertilizer use, livestock waste, manure management, and environmental impacts,” in Encyclopedia of Energy, Natural Resource, and Environmental Economics. ed. Shogren J. F. (Hoboken, New Jersey, U.S.: Elsevier Inc.), 50–57.

[ref31] Jimenez-Lopez C. Fraga-Corral M. Carpena M. García-Oliveira P. Echave J. Pereira A. G.. (2020). Agriculture waste valorisation as a source of antioxidant phenolic compounds within a circular and sustainable bioeconomy. Food Funct. 11, 4853–4877. doi: 10.1039/D0FO00937G, PMID: 32463400

[ref32] Johanningsmeier S. D. Harris G. K. Klevorn C. M. (2016). Metabolomic technologies for improving the quality of food: practice and promise. Annu. Rev. Food Sci. Technol. 7, 413–438. doi: 10.1146/annurev-food-022814-015721, PMID: 26772413

[ref09] John I. Muthukumar K. Arunagiri A. (2017). A review on the potential of citrus waste for D-limonene, pectin, and bioethanol production. Int. J. Green Energy, 14, 599–612. doi: 10.1080/15435075.2017.1307753

[ref33] Kareem S. Rahman R. (2013). Utilization of banana peels for citric acid production by *Aspergillus Niger*. Agric. Biol. J. North Am. 4, 384–387. doi: 10.5251/abjna.2013.4.4.384.387

[ref34] Kennedy D. O. Wightman E. L. (2011). Herbal extracts and phytochemicals: plant secondary metabolites and the enhancement of human brain function. Adv. Nutr. 2, 32–50. doi: 10.3945/an.110.000117, PMID: 22211188 PMC3042794

[ref35] Kraithong S. Issara U. (2021). A strategic review on plant by-product from banana harvesting: A potentially bio-based ingredient for approaching novel food and agro-industry sustainability. J. Saudi Soc. Agric. Sci. 20, 530–543. doi: 10.1016/j.jssas.2021.06.004

[ref36] Long J. M. Mohan A. (2021). Food flavoring prepared with lemon by-product. J. Food Process. Preserv. 45:e15462. doi: 10.1111/jfpp.15462

[ref37] Lu Z. Wang J. Gao R. Ye F. Zhao G. (2019). Sustainable valorisation of tomato pomace: a comprehensive review. Trends Food Sci. Technol. 86, 172–187. doi: 10.1016/j.tifs.2019.02.020

[ref38] Lucarini M. Durazzo A. Romani A. Campo M. Lombardi-Boccia G. Cecchini F. (2018). Bio-based compounds from grape seeds: A biorefinery approach. Molecules 23:1888. doi: 10.3390/molecules23081888, PMID: 30060557 PMC6222734

[ref39] Luna-Vital D. Li Q. West L. West M. De Mejia E. G. (2017). Anthocyanin condensed forms do not affect color or chemical stability of purple corn pericarp extracts stored under different pHs. Food Chem. 232, 639–647. doi: 10.1016/j.foodchem.2017.03.169, PMID: 28490122

[ref40] Mattos G. N. Tonon R. V. Furtado A. A. L. Cabral L. M. C. (2017). Grape by-product extracts against microbial proliferation and lipid oxidation: a review. J. Sci. Food Agric. 97, 1055–1064. doi: 10.1002/jsfa.8062, PMID: 27696415

[ref41] Meng F. Dungait J. A. J. Xu X. Bol R. Zhang X. Wu W. (2017). Coupled incorporation of maize (*Zea mays* L.) straw with nitrogen fertilizer increased soil organic carbon in Fluvic Cambisol. Geoderma 304, 19–27. doi: 10.1016/j.geoderma.2016.09.010

[ref42] Meselhy K. M. Shams M. M. Sherif N. H. El-Sonbaty S. M. (2020). Phytochemical study, potential cytotoxic and antioxidant activities of selected food byproducts (pomegranate peel, Rice bran, Rice straw & mulberry bark). Nat. Prod. Res. 34, 530–533. doi: 10.1080/14786419.2018.1488708, PMID: 30080101

[ref05] Morales-Contreras B. E. Contreras-Esquivel J. C. Wicker L. Ochoa-Martínez L. A. Morales-Castro J. (2017). Husk Tomato (*Physalis ixocarpa Brot.*). Waste as a Promising Source of Pectin: Extraction and Physicochemical Characterization. J. Food Sci. 82, 1594–1601. doi: 10.1111/1750-3841.13768 28585703

[ref43] Nagendran R. (2011). “Agricultural waste and pollution,” in Waste. eds. Letcher T. M. Vallero D. A. (Amsterdam, The Netherlands: Elsevier, Inc.), 341–355.

[ref44] Negro V. Ruggeri B. Fino D. (2018). Recovery of energy from orange peels through anaerobic digestion and pyrolysis processes after d-limonene extraction. Waste Biomass Valorization. 9, 1331–1337. doi: 10.1007/s12649-017-9915-z

[ref45] Nerantzis E. T. Tataridis P. (2006). Integrated enology-utilization of winery by-products into high added value products. E J. Sci. Technol 1, 79–89.

[ref46] Nigam P. S. (2017). An overview: recycling of solid barley waste generated as a by-product in distillery and brewery. Waste Manag. 62, 255–261. doi: 10.1016/j.wasman.2017.02.018, PMID: 28237364

[ref47] Nile S. H. Nile A. Liu J. Kim D. H. Kai G. (2019). Exploitation of apple pomace towards extraction of triterpenic acids, antioxidant potential, cytotoxic effects, and inhibition of clinically important enzymes. Food Chem. Toxicol. 131:110563. doi: 10.1016/j.fct.2019.110563, PMID: 31199992

[ref48] Otify A. M. El-Sayed A. M. Michel C. G. Farag M. A. (2019). Metabolites profiling of date palm (*Phoenix dactylifera* L.) commercial by-products (pits and pollen) in relation to its antioxidant effect: a multiplex approach of MS and NMR metabolomics. Metabolomics 15:119. doi: 10.1007/s11306-019-1581-7, PMID: 31456052

[ref49] Pacheco M. T. Moreno F. J. Villamiel M. (2018). Chemical and physicochemical characterization of orange by-products derived from industry. J. Sci. Food Agric. 99, 868–876. doi: 10.1002/jsfa.9257 30009444

[ref50] Patti G. J. Yanes O. Siuzdak G. (2012). Innovation: metabolomics: the apogee of the omics trilogy. Nat. Rev. Mol. Cell Biol. 13, 263–269. doi: 10.1038/nrm3314, PMID: 22436749 PMC3682684

[ref06] Panpetch P. Sirikantaramas S. (2021). Fruit ripening-associated leucylaminopeptidase with cysteinylglycine dipeptidase activity from durian suggests its involvement in glutathione recycling. BMC Plant Biology. 21, 1–14. doi: 10.1186/s12870-021-02845-6 33526024 PMC7852106

[ref51] Peanparkdee M. Iwamoto S. (2019). Bioactive compounds from by-products of rice cultivation and rice processing: extraction and application in the food and pharmaceutical industries. Trends Food Sci. Technol. 86, 109–117. doi: 10.1016/j.tifs.2019.02.041

[ref52] Perez-Ternero C. De Sotomayor M. A. Herrera M. D. (2017). Contribution of ferulic acid, γ-oryzanol and tocotrienols to the cardiometabolic protective effects of rice bran. J. Funct. Foods 32, 58–71. doi: 10.1016/j.jff.2017.02.014

[ref07] Pinsorn P. Oikawa A. Watanabe M. Sasaki R. Ngamchuachit P. Hoefgen R.. (2018). Metabolic variation in the pulps of two durian cultivars: Unraveling the metabolites that contribute to the flavor. Food Chem. 268, 118–125. doi: 10.1016/j.foodchem.2018.06.066, PMID: 30064738

[ref53] Putri S. P. Nakayama Y. Matsuda F. Uchikata T. Kobayashi S. Matsubara A.. (2013). Current metabolomics: practical applications. J. Biosci. Bioeng. 115, 579–589. doi: 10.1016/j.jbiosc.2012.12.007 23369275

[ref08] Quispe I. Navia R. Kahhat R. (2017). Energy potential from rice husk through direct combustion and fast pyrolysis: a review. Waste Manag. 59, 200–210. doi: 10.1016/j.wasman.2016.10.001 27751683

[ref54] Ramankutty N. Mehrabi Z. Waha K. Jarvis L. Kremen C. Herrero M.. (2018). Trends in global agricultural land use: implications for environmental health and food security. Annu. Rev. Plant Biol. 69, 789–815. doi: 10.1146/annurev-arplant-042817-040256, PMID: 29489395

[ref55] Ramli A. N. M. Sukri B. M. Azelee N. A. W. Bhuyar P. (2021). Exploration of antibacterial and antioxidative activity of seed/peel extracts of south-east Asian fruit durian (*Durio zibethinus*) for effective shelf-life enhancement of preserved meat. J. Food Process. Preserv. 45:e15662. doi: 10.1111/jfpp.15662

[ref56] Rockström J. Williams J. Daily G. Noble A. Matthews N. Gordon L.. (2017). Sustainable intensification of agriculture for human prosperity and global sustainability. Ambio 46, 4–17. doi: 10.1007/s13280-016-0793-6, PMID: 27405653 PMC5226894

[ref010] Roda A. Lucini L. Torchio F. Dordoni R. De Faveri D. M. Lambri M. (2017). Metabolite profiling and volatiles of pineapple wine and vinegar obtained from pineapple waste. Food Chem. 229, 734–742. doi: 10.1016/j.foodchem.2017.02.111 28372238

[ref57] Rubert J. Zachariasova M. Hajslova J. (2015). Advances in high-resolution mass spectrometry based on metabolomics studies for food-a review. Food Addit. Contam. Part A Chem. Anal. Control Expo. Risk Assess. 32, 1685–1708. doi: 10.1080/19440049.2015.1084539, PMID: 26365870

[ref58] Sandhya S. Khamrui K. Prasad W. Kumar M. Kumar C. M. (2018). Preparation of pomegranate peel extract powder and evaluation of its effect on functional properties and shelf life of curd. LWT. 92, 416–421. doi: 10.1016/j.lwt.2018.02.057

[ref59] Scalbert A. Brennan L. Fiehn O. Hankemeier T. Kristal B. S. Van Ommen B. (2009). Mass-spectrometry-based metabolomics: limitations and recommendations for future progress with particular focus on nutrition research. Metabolomics 5, 435–458. doi: 10.1007/s11306-009-0168-0, PMID: 20046865 PMC2794347

[ref012] Sampaio S. L. Petropoulos S. A. Alexopoulos A. Heleno S. A. Santos-Buelga C. Barros L.. (2020). Potato peels as sources of functional compounds for the food industry: a review. Trends Food Sci. Technol. 103, 118–129. doi: 10.1016/j.tifs.2020.07.015

[ref013] Sangpong L. Khaksar G. Pinsorn P. Oikawa A. Sasaki R. Erban A.. (2021). Assessing dynamic changes of taste-related primary metabolism during ripening of durian pulp using metabolomic and transcriptomic analyses. Front. Plant Sci. 12:687799. doi: 10.3389/fpls.2021.687799 34220909 PMC8250156

[ref60] Scharf R. Wang R. Maycock J. Ho P. Chen S. Orfila C. (2020). Valorisation of potato (*Solanum tuberosum*) peel waste: extraction of fibre, monosaccharides, and uronic acids. Waste Biomass Valor. 11, 2123–2128. doi: 10.1007/s12649-018-0532-2

[ref61] Seifdavati J. Seifzadeh S. Ramezani M. Mashak R. B. Seyedsharifi R. Elghandour M. M. M. Y.. (2021). Wastes valorization of wheat straw and wheat bran treated with urea, probiotic or organic acids to enhance ruminal gas production and digestibility of pumpkin by-product. Waste Biomass Valor. 12, 5979–5989. doi: 10.1007/s12649-021-01432-y

[ref62] Serrano A. Fermoso F. G. Alonso-Fariñas B. Rodríguez-Gutierrez G. Fernandez-Bolaños J. Borja R. (2017). Olive mill solid waste biorefinery: high-temperature thermal pretreatment for phenol recovery and biomethanization. J. Clean. Prod. 148, 314–323. doi: 10.1016/j.jclepro.2017.01.152

[ref63] Sharma K. Mahato N. Cho M. H. Lee Y. R. (2017). Converting citrus wastes into value-added products: economic and environmently friendly approaches. Nutrition 34, 29–46. doi: 10.1016/j.nut.2016.09.006, PMID: 28063510

[ref64] Siddique S. Nawaz S. Muhammad F. Akhtar B. Aslam B. (2018). Phytochemical screening and *in-vitro* evaluation of pharmacological activities of peels of *Musa sapientum* and *Carica papaya* fruit. Nat. Prod. Res. 32, 1333–1336. doi: 10.1080/14786419.2017.1342089, PMID: 28627245

[ref65] Singh K. Kumar T. Prince V. K. Sharma S. Rani J. (2019). A review on conversion of food wastes and by-products into value added products. IJCS 7, 2068–2073.

[ref66] Sisti L. Gioia C. Totaro G. Verstichel S. Cartabia M. Camere S.. (2021). Valorization of wheat bran agro-industrial byproduct as an upgrading filler for mycelium-based composite materials. Ind. Crop. Prod. 170:113742. doi: 10.1016/j.indcrop.2021.113742

[ref67] Sud D. Mahajan G. Kaur M. P. (2008). Agricultural waste material as potential adsorbent for sequestering heavy metal ions from aqueous solutions - a review. Bioresour. Technol. 99, 6017–6027. doi: 10.1016/j.biortech.2007.11.064, PMID: 18280151

[ref68] Szabo K. Cătoi A. F. Vodnar D. C. (2018). Bioactive compounds extracted from tomato processing by-products as a source of valuable nutrients. Plant Foods Hum. Nutr. 73, 268–277. doi: 10.1007/s11130-018-0691-0, PMID: 30264237

[ref69] Tremocoldi M. A. Rosalen P. L. Franchin M. Massarioli A. P. Denny C. Daiuto E. R.. (2018). Exploration of avocado by-products as natural sources of bioactive compounds. PLoS One 13:e0192577. doi: 10.1371/journal.pone.0192577, PMID: 29444125 PMC5812635

[ref011] Truzzi E. Chaouch M. A. Rossi G. Tagliazucchi L. Bertelli D. Benvenuti S.. (2022). Characterization and Valorization of the Agricultural Waste Obtained from Lavandula Steam Distillation for Its Reuse in the Food and Pharmaceutical. Fields. Mol. 27:1613. doi: 10.3390/molecules27051613 PMC8911589 35268713

[ref70] Wall-Medrano A. Olivas-Aguirre F. J. Ayala-Zavala J. F. Domínguez-Avila J. A. Gonzalez Aguilar G. A. Herrera-Cazares L. A.. (2020). “Health benefits of mango by-products,” in Food Wastes and By-Products: Nutraceutical and Health Potential. eds. Campos-Vega R. Oomah B. D. Vergara-Castaneda H. A. (Hoboken, New Jersey, U.S.: Blackwell Publishing), 159–191.

[ref71] Wang W. Akhtar K. Ren G. Yang G. Feng Y. Yuan L. (2019). Impact of straw management on seasonal soil carbon dioxide emissions, soil water content, and temperature in a semi-arid region of China. Sci. Total Environ. 652, 471–482. doi: 10.1016/j.scitotenv.2018.10.207, PMID: 30368177

[ref72] Wong Y. S. Sia C. M. Khoo H. E. Ang Y. K. Chang S. K. Yim H. S. (2014). Influence of extraction conditions on antioxidant properties of passion fruit (*Passiflora Edulis*) peel. Acta. Sci. Pol. Technol. Aliment. 13, 257–265. doi: 10.17306/J.AFS.2014.3.4, PMID: 24887941

[ref73] Wurtzel E. T. Kutchan T. M. (2016). Plant metabolism, the diverse chemistry set of the future. Science 353, 1232–1236. doi: 10.1126/science.aad2062, PMID: 27634523

[ref74] Xu Y. Li Y. Bao T. Zheng X. Chen W. Wang J. (2017). A recyclable protein resource derived from cauliflower by-products: potential biological activities of protein hydrolysates. Food Chem. 221, 114–122. doi: 10.1016/j.foodchem.2016.10.053, PMID: 27979071

[ref014] Yang C. Chen H. Chen H. Zhong B. Luo X. Chun J. (2017). Antioxidant and anticancer activities of essential oil from Gannan navel orange peel. Molecules 22:1391. doi: 10.3390/molecules22081391, PMID: 28829378 PMC6152265

[ref75] Zheng W. Wang S. Y. (2001). Antioxidant activity and phenolic compounds in selected herbs. J. Agric. Food Chem. 49, 5165–5170. doi: 10.1021/jf010697n 11714298

